# White blood cell concentration correlates with increased concentrations of IL-1ra and improvement in WOMAC pain scores in an open-label safety study of autologous protein solution

**DOI:** 10.1186/s40634-016-0043-7

**Published:** 2016-02-09

**Authors:** William King, Walter van der Weegen, Rogier Van Drumpt, Hans Soons, Krista Toler, Jennifer Woodell-May

**Affiliations:** St. Anna Hospital, Geldrop, The Netherland; Biomet, 56 East Bell Drive, Warsaw, IN 46581 USA

**Keywords:** Inflammation, Pain, Function, IL-1ra, Growth factor, Platelet-rich plasma

## Abstract

**Background:**

There has been debate on which blood components should be included in autologous therapies. Autologous Protein Solution (APS) is a unique blood-derived therapy composed of concentrated white blood cells, platelets, and plasma to contain high concentrations of anti-inflammatory cytokines and anabolic growth factors to potentially address osteoarthritis. The primary aim of the exploratory secondary analysis was to identify characteristics of an Autologous Protein Solution (APS) that may correlate with improved Western Ontario and McMaster Universities Osteoarthritis Index (WOMAC) scores and OMERACT-OARSI responder status after treatment of subjects with an intra-articular injection of APS.

**Methods:**

Eleven subjects were enrolled in a pilot study of a single intra-articular injection of APS in subjects with knee osteoarthritis. Two APS kits were processed per patient. The output of the first APS kit was injected intra-articularly. White blood cell (WBC) and cytokine concentrations were measured from the output of the second APS kit. WOMAC surveys were completed at baseline and at follow up visits. Linear regression analyses were performed on the blood components of APS with subject outcomes. Anderson-Darling analysis was used to determine whether the cytokine concentrations in whole blood and APS had a normal distribution. Either paired t-test analyses or Wilcoxon signed-rank analyses were performed for normal and non-parametrically distributed data, respectively.

**Results:**

The WBC concentration in APS was significantly (*p* < 0.05) and strongly (R^2^ > 0.7) correlated with IL-1ra in APS but not significantly correlated with IL-1β. The ratio of IL-1ra to IL-1β in APS was significantly correlated with improved WOMAC pain scores one week and six months post-injection. 85.7 % of subjects whose APS had a IL-1ra:IL-1β ratio greater than 1000 or a WBC count greater than 30 k/μl were OMERACT-OARSI responders six months post-injection.

**Conclusions:**

The correlations between the IL-1ra:IL-1β ratio and WBC concentration in a subject’s APS and their WOMAC pain scores and classification as OMERACT-OARSI responders suggest the potential utility of these characteristics as diagnostic markers. Additional studies are ongoing to determine whether APS is safe and effective and to further evaluate the relationship between APS composition and clinical outcomes.

**Trial Registration:**

(NCT01773226)

**Electronic supplementary material:**

The online version of this article (doi:10.1186/s40634-016-0043-7) contains supplementary material, which is available to authorized users.

## Background

Osteoarthritis (OA) is a debilitating disease driven by multiple inflammatory signaling pathways. There has been significant interest in the development of autologous therapies because they contain growth factors and cytokines which could inhibit numerous inflammatory signaling pathways which may drive the progression of OA (Filardo et al. 2013). Nonetheless, there are uncertainties regarding the effectiveness and associated mechanism of action of autologous therapies for specific indications (Sheth 2012). This has motivated the need for advanced autologous therapies with cellular and molecular profiles tailored to address specific diseases or injuries.

Autologous Protein Solution (APS) is a new autologous therapy under investigation for the treatment of knee OA. APS is prepared from a small volume of blood at the point-of-care. In this class of therapeutics, APS has a unique profile of concentrated white blood cells (WBCs), platelets, and plasma (O’Shaughnessey et al. 2014). WBCs are the main source of interleukin-1 receptor antagonist (IL-1ra) in the body which competitively inhibits inflammatory interleukin-1β (IL-1β) signaling (Jordan et al. 1995). Platelets’ alpha granules contain anabolic growth factors which are important in cartilage repair pathways (Sanchez et al. 2003) and synergistically act with anti-inflammatory cytokines on the nuclear factor kappa-light-chain-enhancer of activated B cells (NF-κβ) pathway (Montaseri et al. 2011). Plasma contains anti-inflammatory cytokines including soluble interleukin-1 receptor antagonist-type II (sIL-1RII) (Arend et al. 1994) and soluble tumor necrosis factor receptor-type I and -type II (sTNF-RI and sTNF-RII) (Arend 2002). The ability of APS to block both IL-1β and tumor necrosis factorα (TNFα) signaling pathways suggests that it may have utility in the treatment of OA despite the failure of other therapies which block single inflammatory cytokines with recombinant inhibitors (Malemud 2010). The unique cellular and cytokine profile of APS has motivated the exploration of its therapeutic potential.

APS has been tested in cell culture, cartilage explant culture, and in horses with naturally occurring OA. On the cellular level, APS was cultured with macrophages in inflammatory environments, and changes in IL-8 production were measured, demonstrating the ability of APS to reduce inflammation in vitro (O’Shaughnessey et al. 2011). In a separate study, chondrocytes were challenged with recombinant human IL-1β or TNFα and reduction in matrix metalloproteinase-13 (MMP-13 or collagenase) production was measured after APS was introduced to the culture (Woodell-May et al. 2011). On the tissue level, cartilage explants were combined with APS in inflammatory environments, and APS was shown to reduce extracellular matrix degradation and stimulate chondrocyte proliferation (Matuska et al. 2013). APS has been tested in a randomized and controlled trial in horses with naturally occurring OA. Changes in lameness were evaluated using force plate analysis, veterinarian evaluations, and owner surveys (Bertone et al. 2014). Finally, the cytokine profile in APS from 105 subjects with OA was characterized (O’Shaughnessey et al. 2014). Together, these data provided evidence to support an open-label trial to evaluate intra-articular injection of APS in subjects with early to moderate knee OA.

It was hypothesized that increasing concentrations of WBC in APS would be correlated with improved WOMAC pain scores. The secondary analysis described in this manuscript identified characteristics of APS that may correlate with improved Western Ontario and McMaster Universities Osteoarthritis Index (WOMAC) scores and Outcome Measures in Rheumatology-Osteoarthritis Research Society International (OMERACT-OARSI) responder status after treatment with an intra-articular injection of the APS. WOMAC pain scores were analyzed in detail because of the central role of pain when evaluating the effectiveness of potential OA therapies.

## Methods

This first-in-human, open-label, single-center (St. Anna Hospital, Geldrop, Netherlands) study evaluated the safety and tolerability of a single dose of APS in subjects with OA of the knee who have failed at least one conservative therapy (NCT01773226, https://clinicaltrials.gov/ct2/show/NCT01773226). In addition to this primary objective, the secondary objective was to explore the effect of a single injection of APS on pain, joint functionality, and stiffness. Eligibility criteria for this study are summarized in Table 1 and exclusion criteria are summarized in Table 2. A total of 11 subjects were enrolled (Institutional Review Board, Medical Ethics Review Committee Number 1304) after submitting their informed consent and the study was conducted in compliance with the Helsinki Declaration. WOMAC surveys were completed at baseline and at follow-up visits throughout the study, and two APS Kits (nSTRIDE Autologous Protein Solution (APS) Kit, Biomet, USA) were processed per patient.

**Table 1:** Inclusion criteria for patients in the open-label study of APS on patients with OA

Inclusion Criteria
Male or female ≥40 years
Willingness and ability to comply with the study procedures and visit schedules and ability to follow verbal and written instructions
A standing radiograph of the knee showing a Kellgren grade of 2 or 3 (within 6 months prior to screening or at screening)
Frequency of knee pain on most days (>15 days) over the last month
Diagnosis of unilateral knee OA according to the American College of Rheumatology (ACR) criteria (clinical and radiological categories) based on an x-ray performed within 6 months prior to screening
Body mass index (BMI) ≤40 kg/m^2^
On the WOMAC LK 3.1 questionnaire, the mean total score of the 5 pain subscale items together must be ≥ 2.0 at screening and at baseline (Day 1 pre-injection)
Failed at least 1 prior conservative OA therapy (physiotherapy and/or simple analgesics)
Signed an IEC-approved ICF
Willingness to abstain from the use of topical pain therapies (e.g., NSAIDs, capsaicin, lidocaine patches, heat patches), intra-articular corticosteroids, and viscosupplementation during the study
Willingness to abstain from systemic pain medications, except for rescue medication (acetaminophen, maximum daily dose ≤ 4 g); also, to abstain from rescue medication for at least 48 h prior to post-screening study visits

**Table 2:** Exclusion criteria for patients in the open-label study of APS on patients with knee OA

Exclusion Criteria
Disease Related Criteria
On day 1 (pre-injection), presence of active infection or abnormal effusion in the knee as noted by a physical examination (e.g., erythema, redness, heat, swelling)
Diagnosed with rheumatoid arthritis (RA), Reiter’s syndrome, psoriatic arthritis, ankylosing spondylitis, chondromalacia, arthritis secondary to other inflammatory diseases (e.g., inflammatory bowel disease [IBD], sarcoidosis, or amyloidosis)
Arthritis of metabolic origin
Ipsilateral hip OA
Untreated traumatic injury of knee
Limited daily activity for reasons other than OA
Presence of surgical hardware or other foreign body in the knee
Prior or concomitant OA treatment-related criteria
Arthroscopy or open surgery in knee within 12 months prior to screening
Intra-articular steroid injections in any joint within 3 months prior to screening
Intra-articular HA in any joint within 6 months prior to screening
Other intra-articular therapy in any joint within 3 months prior to screening
Taking systemic steroids within 2 weeks prior to screening
Planned/anticipated surgery of the knee during the study period
Patient-related criteria:
Any clinically significant results at screening (values or findings outside of normal ranges that were deemed clinically significant by the investigator)
Active or history of malignancy other than non-melanoma skin cancer
Any serious, non-malignant, significant, acute, or chronic medical condition (e.g., diabetes, cardiopathy) or active psychiatric illness that, in the investigator’s opinion, could have compromised patient safety, limited the patient’s ability to complete the study, and/or compromised the objectives of the study
Skin breakdown at the knee where the injection was planned to take place
Pregnant or nursing mothers, or women likely to conceive a child and unwilling to use a reliable form of birth control for the duration of the study
History of drug or alcohol dependence, and/or positive screening results from urine drug and alcohol tests
Used any investigational drug or device within 30 days prior to screening, or 5 half-lives, whichever is longer
Used any investigational biologic within 60 days prior to screening

To process each APS device, 60 ml of anti-coagulated blood (55 ml blood with 5 ml anticoagulant citrate dextrose solution, solution A (ACD-A; Citra Labs, LLC) was processed using the APS Separator to form a cell solution. The cell solution is processed using the APS Concentrator to produce APS. The production of APS requires less than 20 min and is performed at the point-of-care.

The knee was cleaned with an antiseptic, and a 19 gauge needle was advanced percutaneously into the intra-articular space under ultrasound guidance. A luer-lok syringe was connected to the needle, and all of the available joint fluid was aspirated. Without removing the needle from the joint, the syringe containing the APS was attached and all APS was injected. Subjects were advised to not increase physical activity level for two weeks following the injection and to maintain a stable lifestyle throughout the duration of the study. Subjects were also instructed to withhold rescue medication (acetaminophen) for at least 48 h prior to each follow up visit.

WBC and cytokine concentrations (ELISA kits, R&D Systems, Minneapolis, MN) were measured from the output of the second APS Kit as well as a separate whole blood sample. IL-1ra, sIL-1RII, IL-1β, sTNF-RII, and TNFα were measured in this study due to the important and synergistic roles that IL-1β and TNFα play in the progression of OA (Goldring 2000). More information on the ELISA kits can be found in Additional file 1: Table S1. The post-injection severity and frequency of pain was assessed by the patient using the WOMAC LK 3.1 questionnaire at weeks one and two and months one, three, and six post-injection.

Missing data was handled using last observation carried forward (LOCF) principles. The OMERACT-OARSI criteria were used to determine which subjects responded to APS therapy (improvement in pain ≥ 50 % and absolute change > 20) (Pham et al. 2003). WBC analyses were not completed on the APS of the first three subjects, and APS samples were compromised for two subjects. Therefore, these data points were censored leaving nine subjects’ data for cytokine correlation and eight subjects’ data for WBC correlation. Regression analyses were performed using Microsoft Excel 2010 Software. Anderson-Darling analysis was used to determine whether the cytokine concentrations in whole blood and APS had a normal distribution (Minitab Software, State College, PA). If the concentrations across the population had a normal distribution, per Anderson-Darling analysis, then paired t-test analyses were performed. If the concentrations were non-parametric, then Wilcoxon signed-rank analyses were performed. The level of evidence of this study was: Level IV – case series with no comparison group.

## Results

Twenty-one subjects were screened and 11 subjects were enrolled including four females and seven males. One patient withdrew at six weeks and 10 completed the study. Eleven subjects were included in the data set for safety, tolerability, and performance. There were no serious adverse events (SAEs). No AEs were attributed to APS. Treatment with APS resulted in a 72.7 % responder rate (improvement in pain ≥ 50 % and absolute change ≥ 20).

Subjects had an average age of 57.5 ± 9.5 years, a weight of 83.6 ± 10.7 kg, height of 177.1 ± 6.7 cm and a body mass index (BMI) of 26.6 ± 3.1 kg/M^2^. 10 subjects were ethnically Dutch and 1 was Indonesian. The left knee was treated in 54.5 % of subjects and the right knee was treated in 45.5 % of subjects.

Cytokine analysis was performed to measure device performance and to begin to characterize correlation between APS cytokine content and clinical outcomes. The APS kit produced a solution with a greater concentration of cytokines than baseline whole blood (IL-1ra: *p* < 0.001, paired-t test), (IL-1β: *p* = 0.009 Wilcoxon), (sIL-1RII: *p* < 0.001, paired-t test), (sTNF-RII: *p* = 0.009 Wilcoxon), and (TNFα: *p* = 0.036 Wilcoxon) (Table 3). The average ratio of IL-1ra to IL-1β in APS was 8,668 ± 5,600 (IL-1ra_pg/ml_ / IL-1β_pg/ml_) (Table 3).

**Table 3:** Average concentrations of cytokines in whole blood and APS. (average ± standard deviation, *n* = 9)

Metric	IL-1ra	sIL-1RII	IL-1β	sTNF-RII	TNFα
Whole Blood Concentration (pg/ml)	8,029 ± 2,818	6,798 ± 1,623	1.6 ± 0.9	1,036 ± 188	0.7 ± 1.2
APS Concentration (pg/ml)	57,511 ± 24,272	20,121 ± 6,654	20.3 ± 34.2	5,520 ± 1,174	2.6 ± 2.9
Blood Concentration Range (pg/ml)	3,450–11,750	4,622–9,050	0.7–3.3	891–1,563	0.0–3.4
APS Concentration Range (pg/ml)	23,100–97,750	11,350–28,155	2.8–109.3	3,934–7,556	0–7.5
p values	6.8E–05	2.0E–05	0.07	6.2E–07	0.02

The WBC concentration in whole blood and APS had significant and positive correlation (*p* < 0.05) with the concentration of IL-1ra in APS. There was no significant correlation between the concentration of WBC in whole blood or APS and IL-1β (Table 4). Correlation analysis between WBCs and the remaining cytokine concentrations are shown in Additional file 2: Table S2.

**Table 4:** Coefficient of correlation values (R^2^) of whole blood and APS for WBC with IL-1ra and IL-1β

	IL-1ra (pg/ml)		IL-1β (pg/ml)	
WBC (k/μl)	Whole Blood	APS	Whole Blood	APS
	**0.62**	**0.83**	0.06	0.08

*Bold font indicates (*p* < 0.05) and positive correlation. (*n* = 8)

There was no significant correlation between any characteristic of the APS that was measured in this study and subjects’ WOMAC pain scores at the injection visit. The IL-1ra:IL-1β ratios in whole blood and APS were significantly correlated with improved WOMAC pain scores one week (blood *p* = 0.01, APS *p* < 0.01) and six months post-injection (blood *p* = 0.04, APS *p* = 0.04) (Table 5). The WBC concentration (*p* = 0.03) and IL-1ra concentration (*p* = 0.03) in APS were both significantly correlated with improved WOMAC pain scores one week post-injection. The IL-1ra concentration in whole blood (*p* = 0.03) and sTNF-RII concentration in APS (*p* = 0.03) were significantly correlated with improved WOMAC pain scores six months post-injection. The IL-1β concentration in APS was significantly (*p* = 0.045) correlated with diminished WOMAC pain scores six months post-injection, but at no other time points. There was no significant correlation (*p* > 0.05) between the volume of APS administered and WOMAC pain scores at the pre-injection visit, one week post-injection, and six months post-injection (Table 5).

**Table 5:** Coefficient of correlation values (R^2^) of whole blood and APS metrics with WOMAC Pain scores

Metric	Solution	WOMAC Pain-Injection Visit	WOMAC Pain-1 Week	WOMAC Pain-6 Months
WBC Concentration	Whole Blood	0.43	0.42	0.24
	APS	0.01	**0.58**	0.22
IL-1ra:IL-1β Ratio	Whole Blood	0.02	**0.64**	**0.47**
	APS	0.01	**0.37**	**0.46**
IL-1ra	Whole Blood	0.04	0.30	**0.53**
	APS	0.02	**0.52**	0.10
sIL-1RII	Whole Blood	0.19	<0.01	0.02
	APS	0.03	0.32	0.30
IL-1β	Whole Blood	0.01	0.09	0.19
	APS	0.02	0.04	*0.46*
sTNF-RII	Whole Blood	0.02	0.16	0.37
	APS	0.04	0.26	**0.52**
TNFα	Whole Blood	<0.01	0.20	0.38
	APS	0.02	0.39	0.35
Injection Volume	APS	0.03	0.01	0.02

*Bold font represents (*p* < 0.05) and improved pain scores and italicized and underlined font represent significant and diminished pain scores with significant correlation (*n* > 8)

It was also of interest to determine which subjects were more likely to be responders to treatment with APS. The fraction of subjects who had an IL-1ra:IL-1β ratio > 1000 in their APS or had a WBC concentration in their APS of > 30 k/μl had a greater probability of responding to APS therapy than the study population at every time point (Table 6).

**Table 6:** Percent of OMERACT-OARSI responders in different patient populations

% Responders	Week 1	Week 2	Week 4	Month 3	Month 6
Total Population	20.0	45.5	63.6	72.7	72.7
IL-1ra:IL-1β Ratio > 1000	28.6	71.4	71.4	100.0	85.7
WBC > 30 k/μl	28.6	71.4	71.4	100.0	85.7

*Total population (*n* = 11), patients with APS whose IL-1ra:IL-1β ratio > 1000 (*n* = 7/9), and with WBC concentration in APS > 30 k/μl (*n* = 7/8)

## Discussion

APS is an autologous solution with high concentrations of anti-inflammatory cytokines. The ratio of IL-1ra:IL-1β in whole blood and APS was significantly correlated with improved WOMAC pain scores. The subset of subjects whose IL-1ra:IL-1β ratio was > 1000 in their APS had a greater probability of being a OMERACT-OARSI responder than the total study population. Also, subjects with a high concentration of WBCs in their APS demonstrated a greater probability of responding to APS therapy than the total study population. Clinical trials are ongoing to better evaluate the potential utility of these characteristics as potential diagnostic markers to aid in the identification of individuals likely to respond to the APS therapy currently under investigation. The unique cellular and cytokine profile of APS may help explain the correlations observed in this manuscript.

In a previous study in which APS was prepared from 105 subjects with OA, the IL-1ra:Il-1β ratio could be calculated in 98 total subjects due to sample processing errors or the concentration of IL-1β being below the detectable range of the ELISA assay. Of these 98 subjects, 95 had an IL-1ra:IL-1β ratio > 1000 (96.9 %), which represents a conservative measurement due to excluding subjects whose IL-1β concentrations were very low (O’Shaughnessey et al. 2014). In this study, subjects with an IL-1ra:Il-1β ratio > 1000 in their APS had a greater probability of responding to APS therapy than the total study population at every time point. Previously, two APS devices were processed from each of 50 donors, and the WBC concentrations were measured on the 100 outputs. From these outputs, 94 had a WBC concentration > 30 k/μl (94 %) King and Woodell-May (2014a, b) In this study a high percentage of patients had both IL-1ra:IL-1β ratios and WBC concentrations greater than 30 k/μl which is similar to our previous preclinical testing. Therefore, a high percentage of subjects may respond to APS therapy, which would be a desirable trait for any OA therapy. Future testing will be required to better characterize the relationship between the IL-1ra:IL-1β ratio, WBC concentration, and OMERACT-OARSI responder status.

There has been debate on the role of WBCs in autologous therapies. Some in vitro studies suggest that WBCs could promote inflammation and MMP production (Assirelli et al. 2014; Braun et al. 2014; Browning et al. 2012). However, these studies and others have used platelet-rich plasma (PRP) and tissues from unmatched donor sources (Cross et al. 2015), which could induce WBCs to elicit an immune response. Some studies have demonstrated increased concentrations of catabolic factors in WBC-containing autologous products compared to baseline blood; however, these concentrations are still less than 5 pg/ml (Sundman et al. 2011). Concentrations of catabolic factors at these levels are unlikely to have any physical consequence in OA, as there can be approximately an order of magnitude greater concentration of IL-1β in synovial fluid (Schlaak et al. 1996). One potential mechanism by which WBCs may have mediated the positive effect observed in this study is through microparticles from platelets polarizing monocytes in the APS to become M2 pro-healing cells during or after delivery to the joint (Vasina et al. 2011). Also, WBC-containing autologous products have been shown to down-regulate NFκβ, potentially through reducing the expression of cyclooxygenase 2 (COX2) and inducing production of NFκβ inhibitor α (Iκβα) by chondrocytes (Bendinelli et al. 2010). Taken together, there are multiple mechanisms which may explain the correlation between WBC concentration in APS and improvement in WOMAC pain scores. Further investigation will be required to develop a better understanding of the mechanism(s) by which WBC concentrations may impact clinical response to the APS therapy under investigation for the treatment of OA.

The high concentrations of WBCs in APS enable it to have a high ratio of anti-inflammatory cytokines to inflammatory cytokines. Previous studies have demonstrated that WBCs are the primary source of IL-1ra in humans (Jordan et al. 1995) and in APS (King and Woodell-May 2014a, b; King and Woodell-May 2013; King and Woodell-May 2014b). In this study, the ratio of IL-1ra:IL-1β was significantly correlated with improved WOMAC pain scores. The balance between IL-1ra and IL-1β has been shown to play a critical role in the homeostasis of inflammation in diverse diseases in almost every organ system (Arden 2002). IL-1ra:IL-1β ratios of 10–1000 have been shown to inhibit the activity of IL-1β in cell culture and, therefore, have been used as a benchmark for autologous therapies (Wehling et al. 2007). This manuscript provides the first clinical evidence that a IL-1ra:IL-1β ratio of > 1000, in the context of APS, may predict the inhibition of inflammation in OA patients. However, this study included a small number of homogeneous patients and therefore further testing is underway to better characterize these metrics with larger numbers of subjects and more diverse populations. This observation highlights that measuring the concentration of only anti-inflammatory or only inflammatory cytokines in an autologous product may provide an incomplete picture of the processes that balance inflammation.

Although we did not observe a significant correlation between soluble inhibitors of inflammatory cytokines in APS and WOMAC pain scores, they still may have played an underlying role in mediating the activity of APS. Recombinant inhibitors of IL-1β have not yet proven to be effective at treating OA (Chevalier et al. 2013). Therefore, a solution with a high IL-1ra:IL-1β ratio alone may not be sufficient to inhibit OA pain. Although they were not measured in this study, concentrated plasma in APS has high concentrations of anabolic growth factors (O’Shaughnessey et al. 2014). Anabolic growth factors, including platelet derived growth factor (PDGF) and insulin-like growth factor-1 (IGF-1), are concentrated in APS and act in concert to decrease IL-1β-induced NF-κβ activation (Montaseri et al. 2011). The unique cytokine profile in APS may have contributed to the improved WOMAC pain scores in this study.

There were several limitations of this work. As the primary endpoint of this first-in-human study was patient safety, only a relatively small number of subjects were enrolled (*n* = 11), there was no control group, and there was a relatively homogeneous population. The sample size available was further reduced due to incomplete WBC measurements on the first three subjects and improperly stored APS samples on the first two subjects. Current trials are ongoing (NCT02138890 and NCT02262364) to collect data which can be used to further examine the relationships explored in this manuscript.

## Conclusions

This study provided the first evidence that WBC concentration and the ratio of IL-1ra:IL-1β may correlate with improvement in WOMAC pain scores in patients with early OA. More subjects and clinical studies with control groups will be necessary to validate these measurements. These results begin to bridge the preclinical testing of APS with clinical results. Preclinical testing demonstrated that the concentrated platelets, plasma, and white blood cells in APS allow it to inhibit inflammation and reduce cartilage degradation. The results of this study suggest that WBC concentration and the IL-1ra:IL-1β ratio are candidates for further investigation and potential development as diagnostics to predict which subjects are more likely to experience an analgesic effect from APS. Future studies will be required to confirm these findings and to determine whether APS is safe and effective for the treatment of OA.

## Additional files

Additional file 1: Table S1. Information on ELISA assay kits. (DOCX 13.9 kb) Additional file 2: Table S2. Coefficient of correlation values (R^2^) of whole blood and APS for WBC with sIL-1RII, sTNF-RII, and TNFα. (DOCX 16.2 kb)

## Abbreviations

ACD-A: anticoagulant citrate dextrose solution, solution A
APS: Autologous Protein Solution
COX2: cyclooxygenase-2
ELISA: enzyme-linked immunosorbent assay
IGF-1: insulin-like growth factor-1
IL-1: interleukin-1
IL-1ra: interleukin-1 receptor antagonist
IL-8: interleukin-8
Iκβα: inducing production of NFκβ inhibitor alpha
LOCF: last observation carried forward
MMP-13: matrix metalloproteinase-13
NF-κβ: nuclear factor kappa-light-chain-enhancer of activated b cells
OA: osteoarthritis
OMERACT-OARSI: Outcome Measures in Rheumatology- Osteoarthritis Research Society International
PDGF: platelet derived growth factor
PRP: platelet-rich plasma
SAE: serious adverse events
sIL-1RII: soluble interleukin-1 receptor antagonist receptor type II
sTNF-R: soluble tumor necrosis factor receptor
TNFα: tumor necrosis factor alpha
WBC: white blood cells
WOMAC: Western Ontario and McMaster Universities Osteoarthritis Index
