# Supplementary material for: White blood cell concentration correlates with increased concentrations of IL-1ra and improvement in WOMAC pain scores in an open-label safety study of autologous protein solution
Source: J Exp Orthop. 2016 Feb 9;3:9. doi: 10.1186/s40634-016-0043-7 (PMC4747972; doi:10.1186/s40634-016-0043-7)
Supplement: Additional file 1: Table S1. — Information on ELISA assay kits. (DOCX 13.9 kb) [file 40634_2016_43_MOESM1_ESM.docx]

Supplementary Table 1. Information on ELISA assay kits.

| R&D Systems Quantikine ELISA Kit Information | | | | |
| --- | --- | --- | --- | --- |
| Analyte | Part Number | Concentration Range (pg/ml) | Whole Blood Dilution Factor | APS Dilution Factor |
| IL-1ra | DRA00B | 31.2 - 2,000 | 10X | 50X |
| sIL-1RII | DR1B00 | 31.3 - 1,000 | 50X | 50X |
| IL-1β | DLB50 | 3.9 - 125 | 1X | 1X |
| sTNF-RII | DRT200 | 7.8 - 250 | 20X | 20X |
| TNFα | DTA00C | 15.6 - 1000 | 1X | 1X |
