# Supplementary material for: White blood cell concentration correlates with increased concentrations of IL-1ra and improvement in WOMAC pain scores in an open-label safety study of autologous protein solution
Source: J Exp Orthop. 2016 Feb 9;3:9. doi: 10.1186/s40634-016-0043-7 (PMC4747972; doi:10.1186/s40634-016-0043-7)
Supplement: Additional file 2: Table S2. — Coefficient of correlation values (R2) of whole blood and APS for WBC with sIL-1RII, sTNF-RII, and TNFα. (DOCX 16.2 kb) [file 40634_2016_43_MOESM2_ESM.docx]

Supplementary Table 2. Coefficient of correlation values (R^2^) of whole blood and APS for WBC with sIL-1RII, sTNF-RII, and TNFα.

| APS Correlation | sIL-1RII | sTNF-RII | TNFα |
| --- | --- | --- | --- |
| WBC (k/µl) | 0.8 | **0.52** | 0.49 |
| p value | 0.002 | 0.044 | 0.078 |

*Bold font indicates (p < 0.05) and positive correlation. (n = 8)
